# Pathogen-specific epidemiology and clinical trajectories of fungal infections after kidney transplantation: a prospective multicenter cohort study

**DOI:** 10.1186/s12879-026-13558-2

**Published:** 2026-06-08

**Authors:** Iris Schröter, Daniela Schindler, Christian Morath, Lutz Renders, Joachim Andrassy, Nele Kanzelmeyer, Anja Schork, Martin Zeier, Thomas Giese, Claudia Sommerer

**Affiliations:** 1 Department of Nephrology, University Hospital Heidelberg, Im Neuenheimer Feld 162, D-69120 Heidelberg, Germany; 2 Department of Nephrology, Klinikum Rechts der Isar of the Technical University Munich, Munich, Germany; 3 Department of Nephrology and Hypertension, Klinikum Nürnberg, Nuremberg, Germany; 4 Department of General, Visceral and Transplant Surgery, LMU, Munich, Germany; 5 Department of Pediatric Kidney, Liver, Metabolic and Neurological Diseases, Hannover Medical School Children’s Hospital, Hannover, Germany; 6 Department of Internal Medicine IV, Division of Endocrinology, Diabetology and Nephrology, University Hospital Tübingen, Tübingen, Germany; 7 Department of Immunology, University Hospital Heidelberg, Heidelberg, Germany; 8 German Center for Infection Research (DZIF), Braunschweig, Germany; 9 Institute of Transplant Immunology, Hannover Medical School, Hannover, Germany; 10 Department of Kidney, Liver and Metabolic Diseases, Hannover Medical School, Hannover, Germany; 11 Institute of Virology, Hannover Medical School, Hannover, Germany; 12 Department of Nephrology, University Hospital Heidelberg, Heidelberg, Germany; 13 Department of Internal Medicine III, Division of Cardiology, University Hospital Heidelberg, Heidelberg, Germany; 14 Department of Pediatrics, University Children’s Hospital Heidelberg, Heidelberg, Germany; 15 Department of Internal Medicine III, TUM University Hospital rechts der Isar, Technical University Munich, Munich, Germany; 16 Institute for Medical Microbiology, Immunology and Hygiene, Technical University of Munich, Munich, Germany; 17 Institute for Medical Virology and Epidemiology, University Hospital Tübingen, Tübingen, Germany; 18 University Children’s Hospital, University of Tübingen, Tübingen, Germany; 19 Helmholtz Centre for Infection Research, Braunschweig, Germany; 20 Research Unit of Molecular Epidemiology, Helmholtz Zentrum München, Neuherberg, Germany; 21 Hannover Unified Biobank, Hannover Medical School, Hannover, Germany

**Keywords:** Renal transplantation, Infection, Fungal, Mycoses, Cohort study

## Abstract

**Background:**

Fungal infections contribute substantially to morbidity and mortality after kidney transplantation, yet pathogen-specific epidemiology and clinical risk profiles remain incompletely defined. We aimed to characterize incidence, timing, and pathogen-specific risk factors in a contemporary multicenter cohort.

**Methods:**

This prospective study, performed by the German Center for Infection Research, included 1,258 adult kidney transplant recipients across five German centers (2011–2022). Fungal infections were diagnosed using clinical, radiological, and mycological criteria. Cox regression identified pathogen-specific associations. Detailed clinical presentation was additionally analyzed in a predefined Heidelberg subcohort.

**Results:**

The cumulative incidence of fungal infections was 6.7% (95% CI 5.3–8.5). The most frequent pathogens were Candida albicans (40.6%), non-albicans Candida (26.0%), Aspergillus fumigatus (13.5%), and Pneumocystis jirovecii (13.5%). Fever was absent in 79.2% of episodes, and 67.2% had preceding bacterial infections. Pneumocystis jirovecii pneumonia occurred predominantly between October and April, whereas Aspergillus fumigatus infections were observed year-round. ICU stay (HR 5.7, *p* < 0.01) and prolonged hospitalization were associated with Candida albicans. Prior linezolid exposure (HR 3.9, *p* = 0.037), delayed graft function (HR 2.9, *p* = 0.049), and pancreas–kidney transplantation (HR 5.0, *p* = 0.005) were linked with non-albicans Candida. Carbapenem exposure was associated with Pneumocystis jirovecii pneumonia (HR 6.8, *p* = 0.002) and invasive aspergillosis (HR 9.8, *p* < 0.001). Invasive aspergillosis showed the highest mortality (41.7%). In the Heidelberg subcohort (*n* = 495), invasive aspergillosis was diagnosed mainly during inpatient care or shortly thereafter, while Pneumocystis jirovecii pneumonia was primarily identified after outpatient care.

**Conclusion:**

Fungal infections after kidney transplantation show distinct epidemiological patterns with pathogen-specific risk profiles, supporting risk-adapted monitoring and diagnostics.

**Supplementary information:**

The online version contains supplementary material available at 10.1186/s12879-026-13558-2.

## Background

Immunosuppression renders transplant recipients vulnerable to a range of complications, with infections being the leading cause of morbidity and mortality [1–4]. Among these, fungal infections present a particularly challenging issue owing to their severity, diagnostic difficulties, and treatment complexity [5–7]. Fungal infections in renal transplant recipients are often under-recognized despite their potential to cause severe complications. The higher prevalence of bacterial and viral infections in renal transplant patients has historically directed clinical focus toward these pathogens, relegating fungal infections to a lesser priority in research and clinical management. Fungal infections present significant risks to both patient and graft survival, further highlighting the need for targeted research and interventions. Despite the introduction of updated guidelines and new diagnostic tools, global survival rates for fungal infections have not significantly improved [8]. Existing studies have often approached fungal infections in a generalized manner, failing to address the unique challenges faced by renal transplant recipients. Factors such as prolonged surgical procedures, prior infections, extended hospitalizations, and personalized antibiotic regimens created distinct vulnerabilities in this group [9]. Furthermore, the clinical presentation of fungal infections in transplant recipients is often nonspecific, leading to delays in diagnosis and appropriate therapy. Recent systematic data further highlight that traditional fungal diagnostics in transplant patients remain hampered by limited sensitivity and delayed results, while emerging molecular approaches show promise but are not yet validated for routine independent use. This issue is compounded by the relative lack of empirical treatment options compared with bacterial infections, which increases the complexity of management. In parallel, standardized antifungal prophylaxis beyond Pneumocystis jirovecii pneumonia is lacking [10, 11], underscoring the need for refined risk stratification based on real-world epidemiological data.

To address these gaps, we performed a prospective multicenter analysis within the German Center for Infection Research (DZIF) Transplant Cohort to characterize the epidemiology, timing, and pathogen-specific risk factors of fungal infections in a large Central European kidney transplant population. By integrating clinical trajectories with microbiological data, this study aims to provide a contemporary framework to support context-adapted surveillance strategies and targeted prevention in kidney transplant recipients.

## Methods

### Study design

This prospective multicenter study was performed by the German Center for Infection Research (Deutsches Zentrum für Infektionsforschung; DZIF), which involves five leading transplant centers in Germany: University Hospital Hannover, University Hospital and Renal Center Heidelberg, Technical University of Munich (TU Munich), Ludwig Maximilian University of Munich (LMU Munich), and University Hospital Tuebingen.

The study was conducted in accordance with the Declaration of Helsinki and the International Conference on Harmonization Guidelines for Good Clinical Practice, and was approved by the Ethics Committees of the participating centers (Hannover Medical School Nr 6534, Medical Faculty of the University of Heidelberg Nr S-585/2013, Medical Faculty of the TU Munich Nr 5926/13, LMU Munich Nr 380–15, University Hospital Tuebingen Nr327/2014BO1). Written informed consent was obtained from all the participants.

### Setting & study cohort

This study included adult participants from the DZIF cohort who underwent renal transplantation or simultaneous pancreas-kidney transplantation between January 2011 and September 2022. Transplantation was performed according to standard clinical criteria at all participating centers.

Follow-up visits were scheduled at 3, 6, 9, and 12 months post-transplantation and annually thereafter, with additional visits for infectious events. Data were extracted from the DZIF transplant cohort database, which contains detailed demographic, clinical, microbiological, and follow-up information. Baseline and study visit data were extracted from electronic medical records and systematically recorded in a central web-based database by medical professionals. All deaths occurring during follow-up were included in the analysis, including early post-transplant events.

For a predefined subcohort at Heidelberg University Hospital (*n* = 495), additional detailed clinical data were extracted for patients with invasive aspergillosis and Pneumocystis jirovecii pneumonia, including presenting symptoms, laboratory parameters (C-reactive protein, lymphocyte count, lactate dehydrogenase, uric acid), radiological findings, treatment course, and clinical outcomes. These variables were used for descriptive analyses to characterize pathogen-specific clinical presentation and disease trajectories.

Immunosuppressive therapy, as well as prophylaxis and infection surveillance strategies, were based on the current KDIGO guidelines. The standard immunosuppressive regimen was similar in all centers and included calcineurin inhibitors (tacrolimus (Tac) or cyclosporine A (CsA)), mycophenolate sodium or mycophenolate mofetil, and methylprednisolone. (target trough (C0) levels for Tac were 6–9 ng/ml at month 1, 5–8 ng/ml at month 3, and 4–7 ng/ml thereafter for CsA 150–180 ng/ml, 100–150 ng/ml, and 80–120 ng/ml, respectively. Mycophenolic acid (MPA) was provided either as enteric-coated mycophenolate sodium (1.44 g/day) or mycophenolate mofetil (2 g/day). Induction therapy was tailored to the immunological risk using either basiliximab or thymoglobulin.

All prophylaxis protocols included antiviral prophylaxis with valganciclovir for recipients of organs from CMV (Cytomegalovirus) IgG–positive donors for a minimum of three months. Pneumocystis jirovecii pneumonia (PjP) was prevented with trimethoprim-sulfamethoxazole for 6 months (and subsequently treated for acute rejection for six weeks). Four centers used oral anti-Candida prophylaxis during the first 1–3 months or if >20 mg of methylprednisolone was administered. The regimen consisted of oral nystatin in the form of a suspension (Candiohermal) at a dose of 3 × 1 pipette per day and tablets at 3 × 2 tablets per day, administered after meals.

In this study, fungal infections were diagnosed using a multidisciplinary approach, combining clinical evaluation, host factors, and mycological evidence. For suspected cases of pneumonia, fungal diagnostics rely on bronchoalveolar lavage (BAL) samples. The diagnosis of aspergillosis included positive Aspergillus antigen levels in BAL fluid, serum, and/or fungal culture. For PjP, real-time PCR amplification of P. jirovecii DNA from BAL fluid was performed. Cutaneous and mucosal infections were considered superficial infections and excluded.

The primary endpoints were the incidence, etiology, and timing of fungal infections. Secondary endpoints included pathogen-specific risk factors and patient outcomes such as overall mortality, infection-related mortality, need for intensive care, graft function, and hospitalization duration.

The cumulative incidence rates of fungal infections were calculated for each fungal species, and patient demographics, clinical characteristics, and risk factors were analyzed.

### Statistical analysis

Descriptive statistics were used to summarize the baseline characteristics and infection outcomes. Continuous variables are presented as mean ± standard deviation or median with interquartile range (IQRs), depending on their distribution. Categorical variables are expressed as absolute numbers and percentages. Comparisons between patients with and without fungal infections were made using appropriate statistical tests, such as the chi-square test for categorical variables and the Mann-Whitney U test or t-test for continuous variables. Kaplan-Meier survival analysis was used to evaluate 12-month survival rates after fungal infection. Cumulative incidence rates of fungal infections with 95% confidence intervals were estimated using Kaplan-Meier methods, with time from transplantation to first fungal infection as the event of interest and censoring at death, graft failure, loss to follow-up, or end of follow-up. We performed Cox proportional hazard regression analyses to identify pathogen-specific risk factors and analyze associations. The chronology of events was considered by including them as time-dependent covariates. Multivariate analyses of all data (*p* < 0.10) in the univariate analysis were performed to control for confounding factors. Statistical significance was defined as a *p*-value of less than 0.05.

## Results

### Patient characteristics

A total of 1,258 adult renal transplant recipients from the DZIF transplant cohort were enrolled. Patient numbers per center were as follows: Heidelberg (*n* = 495), Munich LMU (*n* = 233), Munich TU (*n* = 246), Tuebingen (*n* = 146), and Hannover (*n* = 97).

The patients’ characteristics are presented in Table 1. The mean age at the time of transplantation was 51 years (±14 years, range 18 - 80), with 64.4% being male recipients. A total of 65.4% of patients received an allograft from a deceased donor. The median surveillance time was 1242 days (IQR = 596 -1816), amounting to 4,262 patient-years. 1061 recipients were followed up for at least one year.

**Table 1:** Patients demographics

	All		Patients with fungal infection		Patients without fungal infection		*p*
**Total no. of patients**	1258		73		1185		
Demographics							
age at tx (mean ± SD, range)	51 ± 14 (18–80)	1258	59 ± 11 (31–76)	73	50 ± 14 (18–80)	1185	**<0.01**
<50 years	41.5 (522)		17.8 (13)		43.0 (509)		**<0.01**
50 - 65 years	42.5 (535)		49.3 (36)		42.1 (499)		0.272
>65 years	16.0 (201)		32.9 (24)		14.9 (177)		**<0.01**
male gender	64.4 (795)	24	61.1 (44)	72	64.6 (751)	1162	0.612
**Clinical Data**							
Cause of ESRD		1237		72		1165	
Glomerulonephritis	32.2 (398)		20.8 (15)		32.9 (383)		0.827
ADPKD	14.5 (179)		16.7 (12)		14.3 (167)		0.602
Diabetes mellitus	9.3 (115)		15.3 (11)		8.9 (104)		0.136
Nephrosclerosis	4.7 (58)		9.7 (7)		4.4 (51)		0.580
Interstinal nephritis	3.1 (38)		5.6 (4)		2.9 (34)		0.667
Vasculitis and collagenoses	2.9 (36)		2.7 (2)		2.9 (34)		0.949
urologically caused diseases	2.1 (25)		0.0 (0)		2.1 (25)		0.604
other hereditary diseases	5.5 (68)		2.8 (2)		5.7 (66)		0.359
other	25.9 (320)		26.4 (19)		25.8 (301)		0.577
body mass index (mean ± SD)	25.5 ± 5	1238	25.4 ± 4	72	25.5 ± 4	1166	0.967
age group		1184		69		1115	
<50 years	30.0 (355)		23.2 (16)		30.4 (339)		0.226
50–65	43.4 (507)		37.7 (26)		43.1 (481)		0.384
>65	27.2 (322)		39.1 (27)		26.4 (295)		**0.018**
ESP	12.5 (148)	1185	27.1 (19)	70	11.6 (129)	1115	**<0.01**
**CMV Serologies**		1143		69		1074	
D+/ R-	22.6 (258)		27.5 (19)		22.2 (239)		0.305
D+/ R+	35.3 (401)		36.2 (25)		35.2 (378)		0.897
D-/ R+	17.6 (201)		14.5 (10)		17.8 (191)		0.624
D-/ R-	24.6 (281)		21.7 (15)		24.8 (266)		0.666
**Type of tx**							
deceased donation	65.4 (816)	1251	82.2 (60)		64.3 (756)	1175	**<0.01**
pancreas-kidney	5.6 (71)	1258	9.6 (7)	73	5.4 (64)	1185	0.111
AB0-Incompatibility	6.8 (80)	1175	5.7 (4)	70	6.9 (76)	1105	0.473
previous transplantation	16.2 (202)	1250	21.9 (16)	73	18.1 (186)	1025	0.188
Immunized tx	20.8 (262)	1258	27.4 (20)	73	20.4 (242)	1185	0.878
**Immunosuppression**							
Induction therapy		1258					
Basiliximab	83.7 (1052)		83.6 (61)	73	83.7 (992)	1185	0.837
Thymoglobuline	16.3 (206)		16.4 (12)		16.3 (193)		0.744
Plasmapheresis	13.0 (164)		16.4 (12)		4.9 (52)		0.371
any conditioning treatment^a^	27.5 (346)		32.9 (24)		27.2 (322)		0.283
Valganciclovir	76.7	1220	77.8 (56)	72	76.5 (879)	1148	0.887
in-patient stay, (Md, IQR)	17 (13–24)	1234	25 (16–37)	72	17 (13–23)	1162	**<0.001**
delayed graft function^b^	20.3 (247)	1214	36.1 (26)	72	19.3 (221)	1142	**0.01**

Data presented as percentages (no.) unless otherwise indicated. Missing values were excluded

Abbreviations: no. = number, SD = standard deviation, ESRD = end stage renal disease, ADPKD = Autosomal dominant polycystic kidney disease, ESP = Eurotransplant Senior program, tx = transplantation, CMV = Cytomegalovirus, D+/- = donor IgG positive/negative, R-/+ = recipient IgG negative

^a^ Conditioning treatment included plasmapheresis, rituximab, and/or other desensitization procedures prior to transplantation

^b^ Delayed graft function was defined as the requirement for dialysis within the first 7 days after transplantation, excluding dialysis performed solely for hyperkalemia

### Mortality and transplant failure

**Fig. 1:**
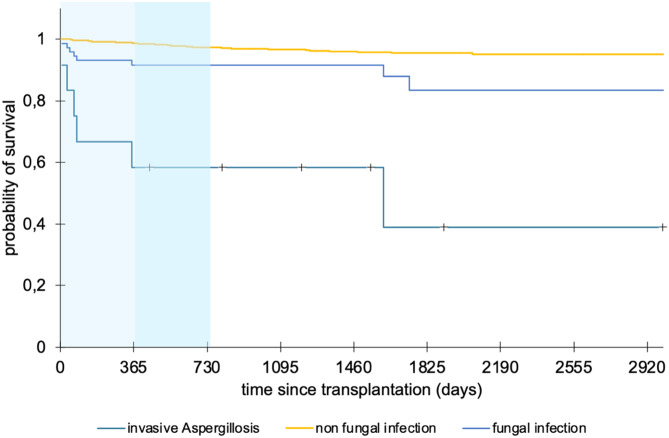
Kaplan–Meier estimates of survival after kidney transplantation stratified by fungal infection status (invasive aspergillosis, other fungal infections, no fungal infection)

Probabilities of survival are visualized in Fig. 1. During the surveillance period, 51 deaths were recorded, resulting in a mortality rate of 5.9% (95% CI: 4.3–7.9). Infection was the leading cause of death (35.5% [18/51]), including six cases of invasive aspergillosis (iA), one disseminated mucormycosis, seven septic-shocks due to bacterial infection with multi-organ failure, two viral infections (Bornavirus encephalitis and SARS-CoV-2 pneumonia), and four cases of unspecified infections. There were 35 cases of transplant failure and 40 cases lost to follow-up. Baseline characteristics did not differ significantly between patients lost to follow-up and those with complete follow-up (all *p* > 0.05). No patients were lost to follow-up within 30 days.

Overall, the predictors of mortality included recipient age > 65 years, deceased donor transplants, participation in the Eurotransplant Senior Program (ESP), delayed graft function (DGF), and prolonged hospital stays of >20 days. Additionally, ICU admissions due to bacterial and fungal infections, especially iA, were also significant contributors to increased mortality risk (Table S1, Supplementary Material).

### Incidence of fungal infections and fungal pathogens

**Fig. 2:**
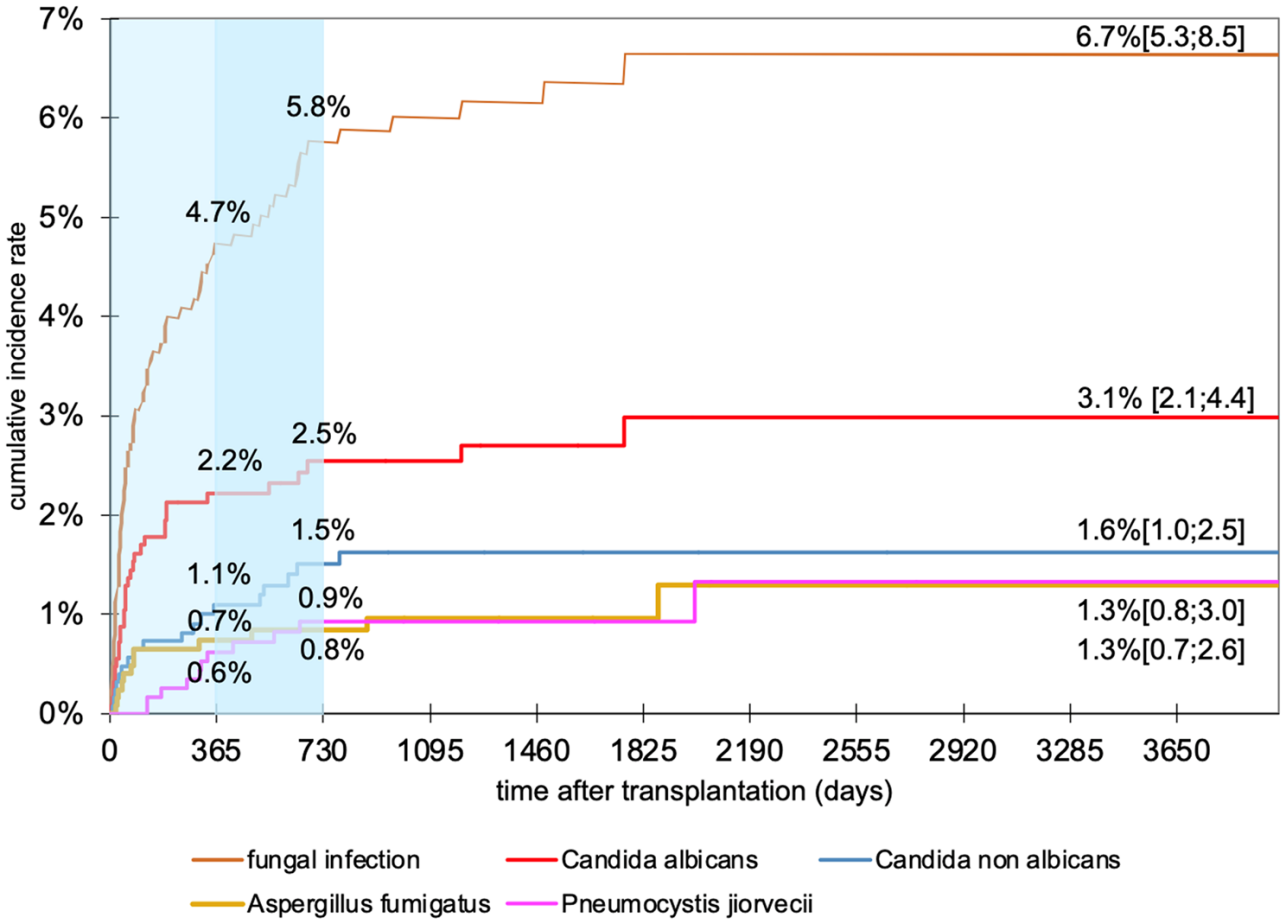
Cumulative incidence rates of the four most commonly isolated fungal pathogens after kidney transplantation

Among 73 patients, 96 fungal infections were identified, yielding a cumulative incidence rate of 6.7% (95% CI: 5.3–8.5). Most patients experienced infections within the first year post-transplant, with a modest increase in the cumulative incidence rate from 4.7% [3.7; 6.1] at the end of the first year to 5.8% [4.6; 7.3] at the end of the second year (Fig. 2). Of the fungal infection episodes, 6.3% were suspected to be related to the origin of the allograft or the site of surgery, predominately caused by Candida species. The most common fungal pathogens were Candida albicans (40.6%), non-albicans Candida species (26.0%), Aspergillus fumigatus (13.5%), and Pneumocystis jirovecii (13.5%). Isolated cases of Aspergillus flavus (pneumonia), Cryptococcus neoformans (fungemia), and Lichtheimia spp. (intra-abdominal mucormycosis with a fatal outcome occurring 8 days post-transplantation).

**Fig. 3:**
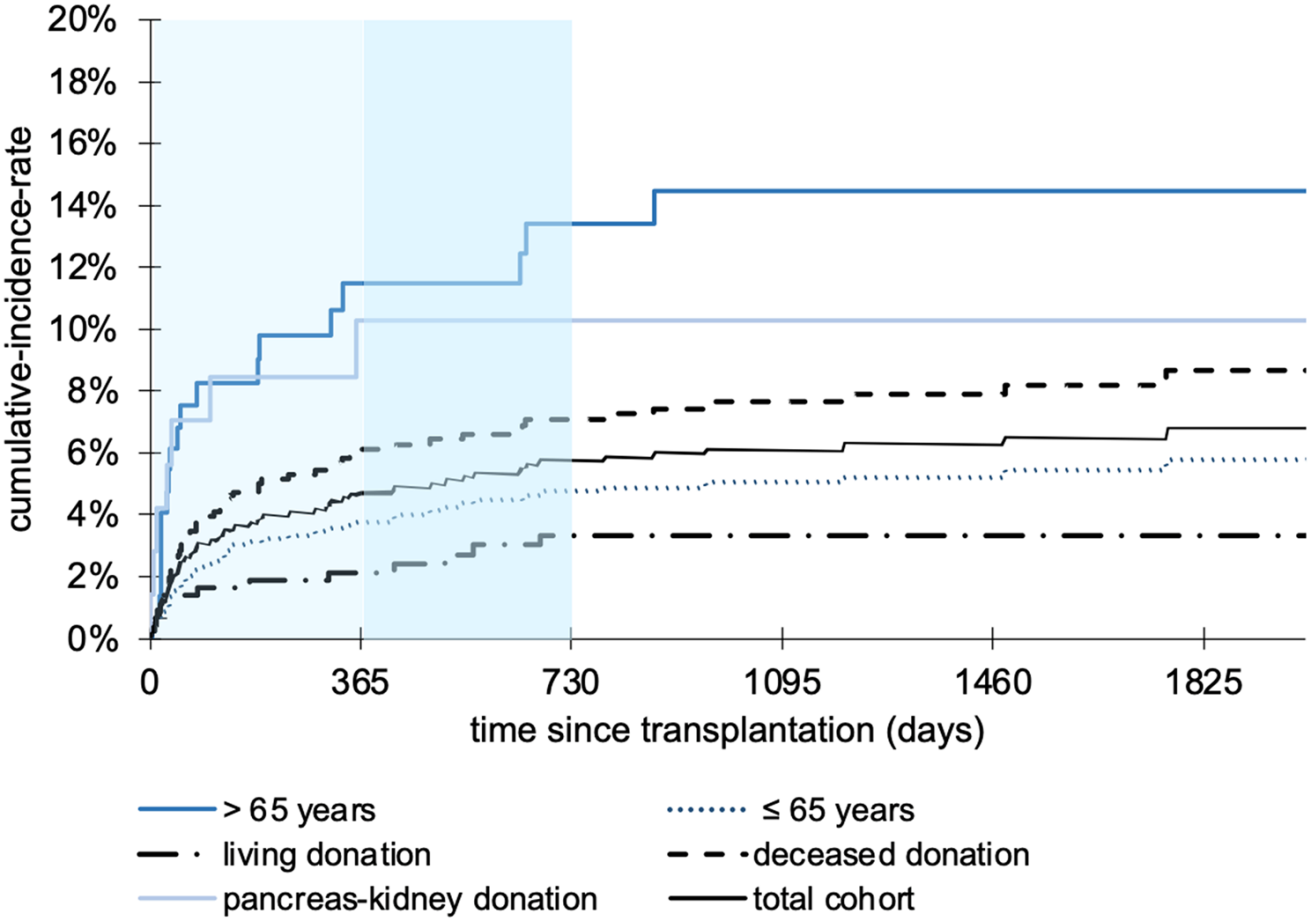
Cumulative incidence rates of fungal infections in DZIF sub-cohorts stratified by recipient age (>65 vs ≤ 65 years) and donor type (living, deceased, pancreas–kidney)

### Clinical features and treatment

Fever (>38.5°C) was absent in 79.2% of the episodes. A total of 19.8% required ICU care, mainly involving catecholamine therapy (78.9%, 15/19). Severe sepsis was diagnosed in 17.7% (17/96) of cases. The overall mortality rate among patients with fungal infections was 16.6% [7.8; 35.0]. Deaths, particularly those due to invasive aspergillosis, occurred early, with a median of 81 days (IQR = 41–635) post-transplant. Common antifungal treatments included fluconazole (25.2%), caspofungin (19.6%), and voriconazole (15.0%). Combination therapy was required for 24 episodes.

### Bacterial infections in patients with fungal infections

A significant proportion (67.2%) of patients with fungal infections had prior bacterial infections, with 8.3% requiring ICU admission, and 23% experiencing bacterial sepsis. The predominant sepsis pathogens were Enterobacter (20.0%), E. coli (20.0%), and Enterococcus spp. (20.0%), with the primary infection sources being the urinary tract (20.0%), surgical site (20.0%), and respiratory tract (20.0%), respectively. Overall, the most frequently isolated pathogen was Enterococcus spp., found in 27.4% of cases, with 46.9% of the strains resistant to vancomycin (VRE). E. coli was present in 20.5% of the cases, with 45.8% of the strains being multi-resistant (3-MRGN). Pseudomonas aeruginosa was identified in 12.0% of the cases, with 71.4% of the strains showing multi-resistance, including both 3-MRGN and 4-MRGN. Pseudomonas aeruginosa was identified as the predominant bacterial pathogen in patients who later developed iA (Table 2b). After fungal infections, the bacterial pathogen spectrum shifted, with an increase in the isolation of Pseudomonas aeruginosa from 12.0% to 17.2%, while the proportion of E. coli decreased from 20.5% to 15.2%. Most patients with fungal infections had previously undergone antibacterial treatment. The predominant antibiotics administered prior to fungal infection were Piperacillin with Tazobactam (14.8%), cephalosporins (14.3%), carbapenems (13.8%), and linezolid (10.7%) (Figure S1, Supplementary Material).

**Table 2b:** Clinical course, co-infections, and outcomes in patients with fungal infections according to pathogen

	Candida albicans	Candida non albicans	Aspergillus fumigatus	Pneumocystis jirovecii
**No. of patients**	33	18	12	13
**Post-operative clinical course**				
in-patient days (no.)	36 (16–49)	28 (20–35)	25 (16–34)	16 (8–24)
Md (IQR), range	10–150	7–128	3–66	3–40
delayed graft function	46.9 (15)	46.7 (7)	33.3 (4)	15.4 (2)
**Renal allograft function month 3**				
impaired function	77.3	50.7	77.8	81.8 (9)
mean eGFR (ml/min/1.73 m^2^)	36.1	53.8	36.7	27.6
**Administered antibacterials prior to fungal infections**				
rate of patients	51.6 (16)	66.7 (12)	66.7 (8)	84.6 (11)
mean no./patient	3.1	4.9	2.2	4.3
**Other infections**				
Bacterial infection				
- any	90.9 (30)	100.0 (18)	69.2 (9)	100 (13)
- preceding	75.8 (25)	77.8 (14)	66.7 (8)	84.6 (11)
- with hospitalization	30.3 (10)	50.0 (90)	61.5 (8)	84.6 (11)
- with ICU admission				
- sepsis				
- with resistant bacteria	54.5 (18)	66.7 (12)	46.1 (6)	38.5 (5)
- VRE	30.3 (10)	55.6 (10)	38.5 (5)	38.5 (5)
− 3MRGN	18.2 (6)	27.8 (5)	23.1 (3)	7.7 (0)
− 4MRGN	3.01 (1)	16.7 (3)	0.0 (0)	0.0 ()
- dominating pathogen	Enterococcus spp. (36.7)	Enterococcus spp. (66.7)	Pseudomonas aeruginosa (44.4)	Enterococcus spp. (30.7)
Viral infection				
- any	48.5 (16)	72.2 (13)	75.0 (9)	53.8 (7)
	BKV (9)	CMV (6)	CMV (5)	CMV (3)
	CMV (5)	HSV-1(2)	HSV-1 (3)	BKV (2)
	HSV-1 (3)	EBV (2)	BKV (2)	HSV-1 (2)
	EBV (3)			
- preceding	21.2 (7)	5.6 (1)	0.0 (0)	16.7 (2)
	BKV (3)	EBV (1)		HSV-1 (1)
	HSV-1 (2)			CMV (1)
	CMV (1)			
	EBV (1)			
**Outcome Data**				
graft failures	3.0 (1)	11.1 (2)	7.7 (1)	7.7 (1)
deaths (any)	12.1 (4)	5.6 (1)	50.0 (6)	7.7 (1)
deaths due to fungal infection	2.9 (1)	5.6 (1)	41.7 (5)	7.7 (1)

Data presented as percentages (no.) unless otherwise indicated. Missing values were excluded

Abbreviations: no. = number, Md = Median, IQR = Interquartile range, eGFR = estimated glomerular filtration rate, spp. = species, ICU = intensive care unit, BKV = BK-Virus, CMV = Cytomegalovirus, HSV-1 = Herpes simplex virus 1, EBV = Epstein-Bar-Virus, VRE = Vancomycin resistant Enterococcus, MRGN = multi resistant gram negative bacteria

### Invasive Aspergillosis and Pneumocystis jirovecii pneumonia

In the overall DZIF cohort, invasive aspergillosis had a median onset of 76 days post-transplantation (IQR 39–351) and a median hospital stay of 19 days (IQR 11–37) (Table 2c). All patients presented with invasive pneumonia, and 41.7% required mechanical ventilation. Voriconazole (*n* = 10), caspofungin (*n* = 7), and amphotericin B deoxycholate (*n* = 4) were the most frequently used antifungal agents. Invasive aspergillosis was associated with the longest treatment duration (median 15 days, IQR 10–20), frequent severe sepsis (53.8%), the highest number of antifungal agents per patient (mean 2.3), and the highest mortality (41.7%). Most deaths (83.3%) occurred within the first three months after transplantation.

**Table 2c:** Characteristics, timing, and antifungal treatment of fungal infection episodes according to pathogen

	Candida albicans	Candida non albicans	Aspergillus fumigatus	Pneumocystis jirovecii
no. of infections	37	26	13	13
timing, d, Md (IQR)				
- first infection	54 (31–188)	100 (96–345)	76 (39–351)	313 (173–422)
- all infections	194 (82–672)	334 (45–642)	81 (42–306)	313 (173–422)
- range	51–1760	7–1656	20–1874	128–1999
- predominating month(s)	month 2	month 1	month 1 + 3	month 4–14
**Antifungal therapy**				
predominating agent (% of treated infections)				
1.	Fluconazole (45.9)	Fluconazole (26.9)	Voriconazole (76.9)	TMP-SMX (100.0)
2.	Caspofungin (21.6)	Caspofungin (15.4)	Caspofungin (53.8)	Caspofungin (15.4)
mean no. /patient	1.1 (1–2)	1.2 (1–2)	2.3 (1–4)	1.6 (1–3)
therapy duration	18 (9–26)	10 (9–13)	15 (10–20)	16 (14–19)
**Clinical course of fungal infection**				
hospital stay (days) Md, IQR	16 (11–27)	11 (7–12)	15 (10–24)	16 (14–19)
ICU admission	10.8 (4)	15.4 (4)	46.2 (6)	23.1 (3)
fever	21.6 (8)	19.2 (5)	28.4 (5)	15.3 (2)
symptoms of sepsis	13.5 (5)	19.2 (5)	53.8 (7)	15.3 (2)
transplant origin	8.1 (3)	7.7 (2)	0.0 (0)	0.0 (0)
**Co-isolations**				
fungal co-isolations	21.6	22.2 (4)	38.5 (5)	30.8 (4)
viral co-isolations requiring antiviral therapy	0.0 (0)	0.0 (0)	30.8 (4) HSV-1(2) CMV (2) HSV-1 + CMV (1) Influenza A (1) (pneumonia)	16.7 (2) HSV-1 (2) (pneumonias)

Data presented as percentages (no.) unless otherwise indicated. Missing values were excluded.

Abbreviations: no. = number, Md = Median, IQR = Interquartile range, ICU = intensive care unit, CMV = Cytomegalovirus, HSV-1 = Herpes simplex virus 1

Pneumocystis jirovecii pneumonia (PjP) occurred later, with a median onset of 313 days post-transplant (IQR 173–422). Most PjP cases (61.5%) developed within the first post-transplant year, with a median hospital stay of 16 days (IQR 13–18). All patients were treated with trimethoprim–sulfamethoxazole. A seasonal pattern was observed, with PjP occurring exclusively between October and April, whereas Aspergillus fumigatus infections were reported throughout the year, with a relative increase during summer months.

Additional detailed clinical data were available for a predefined subcohort at Heidelberg University Hospital (*n* = 495), including 10 patients with 11 episodes of invasive aspergillosis and 7 patients with PjP. Invasive aspergillosis was diagnosed either during inpatient care (54.5%) or shortly after hospitalization (45.5%). Patients typically presented with fatigue and dyspnea, while cough was absent in at least 80% of cases. In contrast, PjP was mainly diagnosed during outpatient follow-up, with patients commonly reporting dry cough and low-grade or subfebrile temperatures. Gastrointestinal symptoms were reported in 42.9% of PjP cases (Figure S2, Supplementary Material). Prior or concurrent herpesvirus infections were documented in 55.5% of patients, predominantly CMV, including cases of CMV pneumonia. Mean BMI was higher in patients with invasive aspergillosis (30 ± 3 kg/m^2^), and diabetes mellitus was present in 36.4%, whereas none of the PjP patients had diabetes. Laboratory findings overlapped, including elevated CRP and lymphopenia, but initial CRP levels were higher in invasive aspergillosis (114 vs. 38 mg/dL). LDH was elevated in all invasive aspergillosis cases, and higher uric acid levels were observed in some patients with PjP (Figure S2, Supplementary Material). Complications of invasive aspergillosis were severe, including graft function decline (64%), respiratory insufficiency requiring mechanical ventilation (40%), sepsis (40%), graft or liver failure (18% each), and a high mortality rate of 54% (Figure S2, Supplementary Material). Causes of death in iA cases included septic dissemination (50%), respiratory failure (33.3%), and meningitis with mycotic basilar aneurysms (16.7%). Most deaths occurred in early onset iA cases, with a median time to death of 74 days (range, 41–288 days). Complications in PjP included graft function decline (71%) and graft failure (14%); however, no PjP-related deaths were recorded (Figure S3, Supplementary Material). All patients with PjP received prophylaxis, with 28.6% on dapsone and 71.4% on trimethoprim/sulfamethoxazole.

### Fungal infections in Eurotransplant Senior Program (ESP) patients

In ESP patients, the cumulative incidence of fungal infections was nearly three times higher than that in non-ESP patients (14.5% [95% CI: 9.5–22.1] vs. 5.8% [95% CI: 4.3–7.6], *p* < 0.001) and almost five times higher than that in recipients of living donor allografts (3.3% [95% CI: 1.9–5.7], *p* < 0.001) (Fig. 3). This difference was most pronounced for Aspergillus fumigatus, where the cumulative incidence rate was ten times higher in ESP patients (7.3% [95% CI: 2.3–23.7] vs. 0.7% [95% CI: 0.3–1.5], *p* = 0.001). ESP patients also developed fungal infections significantly earlier, with a median onset of 48 days (range: 20–251 days) post-transplant, compared to 112 days (range: 37–389 days) in non-ESP patients. Aspergillus fumigatus was the most frequent fungal pathogen observed among ESP recipients in this cohort.

## Risk factors for fungal infections

### Univariate analysis

In univariate analysis, several variables were associated with an increased occurrence of fungal infections (Table 3). Older recipient age was significantly associated with fungal infection (HR = 1.05, 95% CI: 1.030–1.073, *p* < 0.001), as was participation in the Eurotransplant Senior Program (HR = 2.82, 95% CI: 1.68–4.74, *p* = 0.001). Additional factors associated with fungal infection included deceased donor transplantation (HR = 2.47, 95% CI: 1.36–4.50, *p* = 0.001), delayed graft function (HR = 2.54, 95% CI: 1.566–11.9, *p* < 0.001), prolonged postoperative hospitalization (HR = 1.02 per day, 95% CI: 1.018–1.03, *p* < 0.001), ICU admission due to bacterial infection (HR = 7.48, 95% CI: 4.24–13.29, *p* < 0.001), prior linezolid therapy (HR = 3.08, 95% CI: 1.324–3.3329, *p* = 0.002), and lower eGFR at three months post-transplant (HR = 0.981, 95% CI: 0.966–0.907, *p* = 0.021).

**Table 3:** Risk factors of fungal infections (univariate analysis)

	Fungal infection			Candida albicans			Candida non albicans			Pneumocytis jirovecii			Aspergillus fumigatus		
Covariates	HR	95%-CI	p	HR	95%-CI	p	HR	95%-CI	p	HR	95%-CI	p	HR	95%-CI	p
**Baseline Data**															
age at tx	1.051	1.030;1.073	**<0.001**	1.046	1.015;1.077	**0.003**	1.024	0.988;1.061	0.194	1.084	1.021;1.150	**0.008**	1.091	1.029;1.157	**0.004**
male gender	0.844	0.524;1.358	0.485	0.623	0.311;1.247	0.181	0.543	0.216;1.368	0.195	5.483	0.702;42.833	0.105	2.774	0.608;12.662	0.188
ESRD diabetes mellitus	1.806	0.951;3.433	**0.071**	1.023	0.311;3.357	0.971	3.837	1.368;10.764	**0.011**	0.978	0.125;7.641	0.983	1.959	0.429;8.943	0.385
no. of previous tx	1.263	0.853;1.869	0.244	1.203	0.655;2.208	0.551	0.871	0.321:2.434	0.792	1.589	0.688;3.672		0.867	0.245;3.063	0.824
pancreas-kidney tx	1.935	0.887;4.221	0.097	2.482	2.482;7.078	**0.089**	5.003	1.646;15.204	**0.005**	-	-	-	-	-	
**Donor Data**															
deceased donation	2.472	1.356;4.507	**0.003**	2.930	1.128;7.610	**0.027**	1.867	0.615;5.673	0.271	2.525	0.545;11.709	0.237	6.033	0.778;46.756	0.085
ESP	2.819	1.675;4.743	**0.001**	2.621	1.172;5.862	**0.019**	1.581	0.454;5.501	0.472	3.585	1.100;11.685	**0.034**	5.428	1.719;17.138	**0.004**
CMV D+/R-	1.351	0.795;2.294	0.266	1.206	0.539;2.696	0.646	1.089	0.355;3.340	0.882	3.039	0.927;9.964	0.067	1.182	0.320;4.366	0.802
age class donor^1^	1.390	1.011;1.911	**0.043**	1.198	0.751;1.911	0.449	1.173	0.626;2.197	0.619	2.289	0.931;5.628	0.071	2.072	0.891;4.815	0.090
**Induction therapy**															
intensified induction therapy^2^	1.324	0.881;2.161	0.262	1.596	0.780;3.265	0.201	1.324	0.497;3.527	0.575	2.265	0.691;7.431	0.177	0.895	0.242;3.308	0.868
PPH	1.060	0.967;1.161	0.212	1.068	0.937;1.217	0.324	1.072	0.902;1.273	0.431	0.842	0.777;1.363	0.842	0.512	0.084;3.108	0.467
Thymoglobuline	0.992	0.805;1.223	0.942	0.921	0.639;1.327	0.659	1.134	0.840;1.532	0.412	0.766	0.318;1.847	0.553	0.734	0.302;1.768	0.496
**Post-operative course**															
in-patient-days (per day)	1.024	1.018;1.030	**<0.001**	1.027	1.020;1.035	**<0.001**	1.028	1.018;1.038	**<0.001**	1.005	0.968;1.044	0.779	1.018	0.998;1.037	0.073
dgf	2.540	1.566;4.119	**<0.001**	4.048	1.999;8.197	**<0.001**	4.403	1.746;11.103	**0.002**	1.006	0.217;4.659	0.994	2.138	0.643;7.108	0.215
ICU admission due to bacterial infection	7.489	4.240;13.229	**<0.001**	9.180	4.117;20.468	**<0.001**	10.219	3.640;28.691	**<0.001**	5.093	1.125;23.046	**0.035**	5.262	1.147;24.138	**0.033**
**Antibiotic therapy (prior to fungal infection)**															
any	2.099	1.324;3.329	**0.002**	1.739	0.869;3.478	0.118	4.412	1.572;12.380	**0.005**	9.191	2.035;41.506	**0.004**	3.528	1.061;11.725	**0.040**
**Most commonly administered antibiotics of last resort (prior to fungal infection)**															
Linezolid	3.085	1.535;6.199	**0.002**	4.000	1.540;10.392	**0.004**	8.330	2.969;23.372	**<0.001**	3.967	0.877;17.954	0.074	1.971	0.254;15.281	0.516
Carbapenems	1.393	0.693;2.800	0.351	1.022	0.311;3.356	0.971	1.220	0.281;5.308	0.791	6.251	2.043;19.125	**0.001**	**10.134**	3.268;31.429	**<0.001**
**Renal function**															
GFR month 3	0.981	0.907;0.966	**0.021**	0.966	0.941;0.991	**0.009**	1.009	0.985;1.033	0.473	0.950	0.908;0.994	**0.026**	0.968	0.929;1.008	0.114

Univariate Cox proportional hazards models evaluating associations between baseline characteristics, donor factors, perioperative course, antibiotic exposure, renal function, and the occurrence of overall fungal infection as well as pathogen-specific infections (Candida albicans, Candida non-albicans, Pneumocystis jirovecii, and Aspergillus fumigatus)

Hazard ratios (HRs) with 95% confidence intervals (CI) and corresponding *p* values are shown

Abbreviations: HR = Hazard ratio, 95%-CI = 95%-confidence-interval, no. = number, tx = transplantation, ESRD = End stage renal disease, ESP = Eurotransplant Senior Program, CMV = Cytomegalovirus, D+/R- = Donor positive/Recipient negative, PPH = plasmapheresis, ICU = intensive care unit

^1^Donor age was categorized as <50, 50–65, and >65 years

^2^Intensified induction therapy included the use of T-cell depleting agents (e.g., thymoglobulin) and/or additional immunosuppressive agents beyond standard induction

Patients who developed fungal infections were older, with a mean age of 59 ± 11 years at diagnosis. Age distributions differed by pathogen: patients with non-albicans Candida infections had a mean age of 56 ± 6 years, whereas those with invasive aspergillosis had a mean age of 64 ± 8 years (Table 2a). Older age was associated with Aspergillus fumigatus infection and PjP, but not with non-albicans Candida. Pancreas–kidney transplantation was associated with non-albicans Candida infections but not with other fungal pathogens. Prolonged hospitalization and delayed graft function were associated with Candida spp. infections. CMV D+/R− serostatus showed a trend toward association with PjP (HR = 3.039, 95% CI: 0.927–9.964, *p* = 0.067).

**Table 2a:** Baseline

	Candida albicans	Candida non albicans	Aspergillus fumigatus	Pneumocystis jirovecii
**no. of patients**	33	18	12	13
cumulative incidence-rate (%)	3.1	1.6	1.6	1.6
[95%-CI]	[2.1;4.4]	[1.0;2.5]	[0.7;2.6]	[0.8;3.0]
recurrent episodes	6.1 (2)	16.7 (3)	7.7 (1)	0.0 (0)
**Baseline Data**				
age at diagnosis (mean ± SD)	59 ± 6	56 ± 6	64 ± 8	62 ± 6
- <50	24.2 (8)	33.3 (5)	8.3 (1)	0.0 (0)
− 50–65	35.5 (11)	66.7 (10)	41.7 (5)	53.8 (7)
- >65	45.2 (14)	20.0 (3)	50.0 (6)	46.2 (6)
male gender	51.5 (17)	46.7 (7)	83.3 (10)	84.6 (11)
BMI (kg/m^2^)	25.2	25.2	27.1	26.2
predominating cause of ESRD	nephrosclerosis (18.2)	diabetic nephropathy (28.6)	GN (25.0) ADPKD (25.0)	GN (38.5)
deceased donation	84.8 (28)	73.3 (11)	91.7 (11)	76.9 (10)
prior tx	21.2 (7)	13.3 (2)	16.7 (2)	15.4 (2)
pancreas-kidney tx	12.1 (4)	13.3 (2)	0.0 (0)	0.0 (0)
CMV D+/R-	25.0 (8)	23.5	25.0	46.2 (6)
age class donor				
- <50 years	28.2 (9)	23.5 (4)	18.2 (2)	8.3 (1)
− 50–65 years	34.4 (11)	41.2 (7)	27.3 (3)	41.7 (5)
- >65 years	37.5 (12)	35.3 (6)	54.5 (6)	50.0 (6)
AB0 incompatibility	3.0 (1)	9.1 (1)	0.0 (0)	7.7 (1)
intensified induction therapy	36.4 (12)	33.3 (5)	25.0 (3)	38.5 (5)

Data presented as percentages (no.) unless otherwise indicated. Missing values were excluded.

Abbreviations: no. = number, 95%-CI = 95%-confidence-interval, SD = standard deviation, BMI = body mass index, ESRD = end stage renal disease, GN = glomerulonephritis, ADPKD = Autosomal dominant polycystic kidney disease, tx = transplantation, CMV = Cytomegalovirus, D+/R- = Donor IgG positive/Recipient IgG negative

Prior linezolid exposure was associated with both Candida albicans and non-albicans Candida, whereas prior carbapenem use was associated with Aspergillus fumigatus infection and PjP.

### Multivariate analysis

In multivariate analysis, older age at transplantation remained independently associated with fungal infection (HR = 1.044 per year, 95% CI: 1.011–1.077; *p* = 0.008). Prolonged postoperative inpatient stay (HR = 1.038 per day, 95% CI: 1.015–1.062; *p* = 0.001) and prior ICU admission due to severe bacterial infection (HR = 3.639, 95% CI: 1.696–7.810; *p* < 0.01) also remained significantly associated. Previous antibiotic therapy, irrespective of agent class, was independently associated with fungal infection (HR = 2.142, 95% CI: 1.149–3.994; *p* = 0.017). In an alternative multivariable model in which recipient and donor age were replaced by ESP status, ESP remained independently associated with fungal infection. However, overall model performance was inferior compared with the primary age-based model (Supplementary Table S2).

In pathogen-specific models, ICU stay due to bacterial infection (HR = 5.697, 95% CI: 1.961–16.550; *p* < 0.01) and prolonged hospitalization remained associated with Candida albicans. For non-albicans Candida, prior linezolid therapy (HR = 3.910, 95% CI: 1.089–14.040; *p* = 0.037) and delayed graft function (HR = 2.887, 95% CI: 1.005–8.297; *p* = 0.049) remained independently associated.

Carbapenem exposure remained associated with both PjP (HR = 6.848, 95% CI: 2.069–22.664; *p* = 0.002) and invasive aspergillosis (HR = 9.786, 95% CI: 2.610–36.499; *p* < 0.001).

## Discussion

In this large prospective multicenter DZIF cohort of 1,258 renal transplant recipients, we provide a contemporary analysis of fungal infections integrating incidence, timing, and pathogen-specific risk profiles. Our findings demonstrate that fungal infections do not occur randomly after transplantation but arise in distinct clinical contexts characterized by identifiable risk constellations.

The cumulative incidence of 4.8% within the first year is aligns with previous reports, including earlier analyses from our group and the Swiss Transplant Cohort Study [12–16]. However, comprehensive multicenter evaluations capturing the full spectrum of fungal infections in kidney transplant recipients remain scarce, as most prior studies reported fungal disease as composite outcomes. By contrast, our data demonstrate that fungal infections arise in distinct clinical contexts and at different phases after transplantation, emphasizing the heterogeneous nature of post-transplant fungal disease. Consistent with prior renal transplant cohorts, mortality associated with fungal infections was high [17–19], with invasive aspergillosis accounting for most early deaths. In line with Seok et al., older age, deceased donation, and bacterial co-infections were also significant mortality predictors [20]. In our cohort, fungal infections were rarely observed as isolated late events but clustered predominantly within the early post-transplant period, most often in patients with preceding bacterial infection, prolonged hospitalization, or ICU admission. This temporal clustering suggests that invasive fungal disease commonly develops in the setting of early post-transplant clinical instability. Recent longitudinal kidney transplant data similarly demonstrate that invasive fungal infections are associated with a more than threefold increase in mortality and graft failure, with the strongest effect observed within the first six months after transplantation [19]. Complementary DZIF analyses further show that advanced recipient age independently predicts mortality, whereas the infection burden within the first post-transplant year is an independent predictor of both mortality and graft failure [21]. In line with this, fungal infections were significantly more common in older transplant recipients in our cohort [8, 21], further emphasizing the heightened vulnerability of this population during periods of post-transplant instability.Together, these findings indicate a strong association between early infectious complications and adverse outcomes specifically among patients who develop fungal infections after transplantation.

Clinically, this vulnerable phase in our fungal cohort was characterized by severe bacterial infection and critical illness. Prolonged hospitalization and ICU admission due to bacterial sepsis were among the strongest predictors of fungal infection in our cohort, and two-thirds of affected patients had documented bacterial infections beforehand, frequently involving multidrug-resistant organisms. These observations are compatible with a clinically recognizable “two-hit” pattern: patients first develop severe bacterial infection with organ dysfunction requiring ICU care or prolonged hospitalization, and subsequently present with opportunistic fungal disease during the same inpatient episode or shortly thereafter. Such sequential patterns of severe infection followed by opportunistic aspergillosis have also been described in recent analyses of critically ill patient populations [22], supporting the clinical relevance of this trajectory.

This reflects a recurrent bedside scenario in our cohort rather than a mechanistic model and highlights a concrete window in which earlier fungal diagnostics may be particularly relevant. Despite this recurrent inpatient sequence, our data also highlight pathogen-specific clinical patterns, with important exceptions. Candida infections were predominating followed a predominantly nosocomial trajectory, consistent with the substantial clinical burden of candidemia reported in recent global epidemiological analyses [23]. Delayed graft function, prolonged hospitalization, and pancreas–kidney transplantation were key risk factors, reflecting surgical complexity and abdominal disruption. Candida species, commonly commensals, often originate from the intestinal tract of immunosuppressed patients In renal transplant recipients, kidneys are the most heavily colonized organ, with fungal burden correlating with mortality due to tissue damage and potential organ failure [24–29]. Graft preservation solutions may also serve as an additional source for fungal growth, if not adequately tested [30]. Kidney colonization may be underestimated because fungal involvement is not routinely investigated in cases of transplant failure in the absence of obvious clinical signs. Pancreas-kidney transplantation was significantly associated with a higher risk of non-albicans Candida infection, presumably due to the procedural complexity of abdominal disruption. The growing resistance among non-albicans Candida species highlights the need for ongoing surveillance, more precise diagnostic techniques, and new therapeutic strategies to improve patient outcomes [31–33]. In contrast, Pneumocystis jirovecii pneumonia typically presented later during ambulatory follow-up, frequently after completion of prophylaxis, and showed marked seasonal clustering, with most cases occurring during the winter months. This underscores the need for heightened vigilance in outpatient care, particularly during winter, as patients often present with nonspecific respiratory complaints such as dry cough, progressive dyspnea, or fatigue—symptoms that may easily be attributed to viral infections or deconditioning unless PjP is actively considered. Invasive aspergillosis exhibited a distinct inpatient pattern, arising predominantly during acute hospitalization without seasonal variation. Several cases were preceded by Pseudomonas aeruginosa pneumonia with subsequent respiratory deterioration. Respiratory comorbidities and prior severe infections have been identified as important risk factors for secondary invasive fungal infections in critically ill and immunocompromised hosts [34]. Importantly, delayed or missed diagnoses of invasive aspergillosis have been reported, including post-mortem detection [35], further emphasizing the clinical relevance of recognizing these trajectories early. In patients with persistent pulmonary decline despite appropriate antibacterial therapy, early bronchoalveolar lavage and fungal diagnostics may therefore be crucial to shorten time to diagnosis and initiate timely antifungal treatment.

Exposure to broad-spectrum antibiotics, particularly carbapenems and linezolid, was associated with specific fungal pathogens. These associations likely reflect severe preceding infections and antimicrobial pressure rather than direct causal effects, identifying patients who have entered a particularly vulnerable clinical phase.

Despite advances in treatment, such as the incorporation of isavuconazole for mold infections, there remains room for improvement in areas such as the position of combination therapy or the optimal strategy for the reduction of baseline immunosuppression [36–40]. At the same time, systemic antifungal prophylaxis—aside from trimethoprim–sulfamethoxazole for PjP—is not routinely established in renal transplant recipients [11, 12, 41, 42]. Recent surveys highlight wide variation in antifungal prophylaxis practices and underscore lack of standardized evidence in transplant populations [11]. Given the rapid progression of fungal infections, it is critical to identify high-risk patients for tailored prophylaxis or intensified monitoring. A one-size-fits-all approach seems inadequate because fungal infections present unique risk factors, as demonstrated in our findings.

Our study has several strengths, including its prospective multicenter design and detailed clinical characterization of fungal infections in a large transplant cohort. By integrating temporal patterns with pathogen-specific analyses, we provide clinically relevant insights that extend beyond previous studies reporting composite fungal outcomes. Limitations include the non-standardized diagnostic approaches across centers and the limited number of cases for less frequent fungal pathogens. Additionally, center-specific practices and local epidemiology may have influenced the observed associations.

Taken together, our findings argue against uniform fungal surveillance strategies after kidney transplantation. Instead, they support a pathogen- and patient oriented framework. Given the lack of established antifungal prophylaxis and empirical therapy, accompanied by an aging transplant population, further large-scale multicenter studies are urgently needed to stay updated on epidemiological trends and to optimize prophylactic and therapeutic strategies.

## Conclusion

Fungal infections after kidney transplantation follow distinct pathogen-specific clinical trajectories. High-risk constellations, particularly in patients with preceding bacterial infection, ICU stay, and prolonged hospitalization, may help guide risk-adapted monitoring and earlier diagnostic strategies. These findings support a more individualized approach to fungal surveillance in transplant recipients.

## Electronic supplementary material

Below is the link to the electronic supplementary material.

Supplementary Material 1

## Data Availability

The datasets generated and/or analysed during the current study are not publicly available due to patient privacy but are available from the corresponding author on reasonable request.

## Abbreviations

3, MRGN: Multidrug-Resistant Gram-Negative Bacteria (3-drug resistant)
4, MRGN: Multidrug-Resistant Gram-Negative Bacteria (4-drug resistant)
BMI: Body Mass Index
CI: Confidence Interval
CMV: Cytomegalovirus
CRP: C-Reactive Protein
DGF: Delayed Graft Function
eGFR: Estimated Glomerular Filtration Rate
ESP: Eurotransplant Senior Program
HR: Hazard Ratio
ICU: Intensive Care Unit
iA: Invasive Aspergillosis
IQR: Interquartile Range
LDH: Lactate Dehydrogenase
PCR: Polymerase Chain Reaction
PjP: Pneumocystis jirovecii Pneumonia
VRE: Vancomycin-Resistant Enterococci
VZV: Varicella Zoster Virus
