# Supplementary material for: Pathogen-specific epidemiology and clinical trajectories of fungal infections after kidney transplantation: a prospective multicenter cohort study
Source: BMC Infect Dis. 2026 Jun 8;26:1091. doi: 10.1186/s12879-026-13558-2 (PMC13251034; doi:10.1186/s12879-026-13558-2)
Supplement: Supplementary file 1 — Supplementary Material 1 [file 12879_2026_13558_MOESM1_ESM.docx]

**Supplementary Material**

**Table S1.** Univariate associations with all-cause mortality after kidney transplantation

|  | **HR** | **95%-CI** | **p** |
| --- | --- | --- | --- |
| recipient age >65y | 3.333 | 1.854;5.992 | **<0.001** |
| donor age >65y | 3.537 | 1.867;6.721 | **<0.001** |
| deceased donation | 3.051 | 1.093;8.519 | **0.033** |
| ESP | 3.420 | 1.893;6.176 | **<0.001** |
| diabetic nephropathy | 1.248 | 0.646;2.412 | 0.510 |
| in-patient days > 20 | 4.027 | 2.110;7.685 | **<0.001** |
| delayed graft function | 1.834 | 1.002;3.356 | **0.049** |
| antibacterial therapy | 0.653 | 0.360;1.184 | 0.161 |
| ICU admission due to bacterial infection | 6.019 | 2.921;12.402 | **<0.001** |
| fungal infection | 2.793 | 1.511;5.164 | **0.001** |
| Candida albicans | 1.822 | 0.562;5.908 | 0.317 |
| Candida non albicans | 1.440 | 0.347;5.975 | 0.616 |
| Aspergillus fumigatus | 4.896 | 2.050;11.693 | **0.049** |
| Pneumocystis jirovecii | 1.706 | 0.409;7.117 | 0.463 |
| Univariate Cox proportional hazards models assessing associations between recipient and donor characteristics, perioperative factors, infectious complications, and all-cause mortality.  Hazard ratios (HRs) with 95% confidence intervals (CI) and corresponding p values are shown.  Abbreviations: HR = Hazard ratio, 95%-CI = 95% confidence interval, ESP = Eurotransplant Senior Program, ICU = intensive care unit | | | |

**Table S2.** Model performance comparison of multivariable Cox regression models

| **Model** | **Variables** | **C-index (SE)** | **AIC** |
| --- | --- | --- | --- |
| Age-based model | Recipient age (continuous), donor age (categorical), covariates | 0.781 (0.032) | 610 |
| ESP-based model | ESP (instead of age variables), covariates | 0.767 (0.033) | 614 |

**Notes:** Model performance was assessed using Harrell’s concordance index (C-index) and Akaike Information Criterion (AIC). Both models were adjusted for the same covariates (p<0.10 in univariate Cox regression analyses).

**Figure S1**

**Most frequently administered antibacterial agents prior to fungal infection, stratified by fungal pathogen.** Bars indicate the proportion of patients receiving specific antibiotic classes before the diagnosis of Candida albicans, non-albicans Candida, Aspergillus fumigatus, or Pneumocystis jirovecii infection. Percentages above each group indicate the proportion of patients exposed to any antibacterial therapy prior to fungal infection.

**
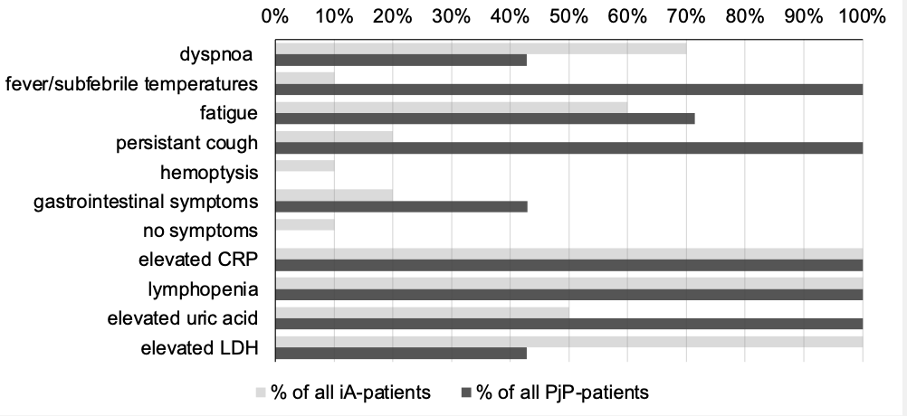
**

**Figure S2.** **Clinical presentation and laboratory abnormalities at diagnosis in renal transplant recipients with invasive aspergillosis (iA) and Pneumocystis jirovecii pneumonia (PjP) within the Heidelberg subcohort (n = 495).**

Bars represent the proportion of patients presenting with individual symptoms or laboratory findings. Light gray bars indicate iA patients, and dark gray bars indicate PjP patients. CRP, C-reactive protein; LDH, lactate dehydrogenase.

**
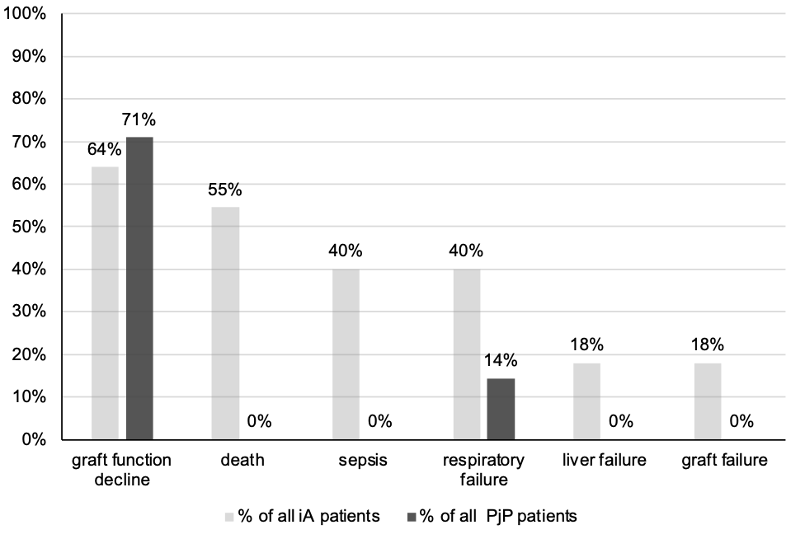
**

**Figure S3.** **Clinical outcomes in renal transplant recipients with invasive aspergillosis (iA) and Pneumocystis jirovecii pneumonia (PjP) within the Heidelberg subcohort (n = 495).**

Bars represent the proportion of patients with individual outcomes, including graft function decline, death, sepsis, respiratory failure, liver failure, and graft failure. Light gray bars indicate iA patients, and dark gray bars indicate PjP patients.
